# Impact of Atrial Fibrillation on the Outcome of Patients with Brugada Syndrome: A Meta-Analysis

**DOI:** 10.3390/jcdd12100391

**Published:** 2025-10-03

**Authors:** Max Aboutorabi, Mahmood Ahmad, Jonathan J. H. Bray, Daniel A. Gomes, Rui Providencia

**Affiliations:** 1 University College London Medical School, Faculty of Medical Sciences, University College London, 74 Huntley St, London WC1E 6DE, UK; max.aboutorabi.19@ucl.ac.uk; 2 Department of Cardiology, Royal Free Hospital, Pond Street, London NW3 2QG, UK; mahmood.ahmad2@nhs.net; 3 Institute of Life Sciences 2, Swansea Bay University Health Board and Swansea University Medical School, Swansea SA2 8PP, UK; jonathanjhbray@gmail.com; 4 Department of Cardiology, Hospital De Santa Cruz, Av. Prof. Dr. Reinaldo dos Santos, 2790-134 Carnaxide, Portugal; 5 Institute of Health Informatics Research, University College London, 222 Euston Road, London NW1 2DA, UK

**Keywords:** Brugada syndrome, atrial fibrillation, major arrhythmic event

## Abstract

Introduction: Atrial fibrillation (AF) is common in patients with Brugada syndrome (BrS). The impact and significance of AF in this patient population needs to be further clarified. Method: We performed a systematic review and meta-analysis of studies comparing the risks of developing major arrhythmic events (MAEs) in patients with BrS with and without AF. Databases including MEDLINE, Embase, and Cochrane CENTRAL were searched from inception to July 2024, using appropriate search and MeSH terms. Data were sought on the comparison of patients with BrS with and without AF. The protocol was specified prior to the searches being performed, and standard meta-analytic techniques were used. Results: Thirteen observational studies were included (a total of 5413 patients). A significant increase in MAEs was observed in patients with both BrS and AF (20.6% vs. 7.8%; OR 2.81, 95% CI 1.82–4.34; *p* < 0.0001; I^2^ = 46%). Significantly higher rates of syncope (33.3% vs. 23.4%; OR 1.97, 95% CI 1.04–3.76; *p* = 0.04, I^2^ = 59%) and a significant increase in all-cause mortality (11.3% vs. 3.7%; OR 4.21, 95% CI 1.69–10.45; *p* = 0.002, I^2^ = 0%) and sodium channel mutations (43.1% vs. 29.9%; OR 1.87, 95% CI 1.07–3.29; *p* = 0.028, I^2^ = 0%) were observed for patients with BrS and AF. Conclusions: Patients with both BrS and AF seem to have a more severe disease phenotype. More research into the added role of AF in risk stratification of asymptomatic BrS patients is needed, but the prognostic implications of AF may need to be considered when developing future personalised medicine approaches in the BrS population.

## 1. Introduction

Brugada syndrome (BrS) is an inherited cardiac condition, first described by Josep and Pedro Brugada, characterised by right bundle branch block and ST-segment elevation in leads V1–V3 that increases a patient’s risk of ventricular arrhythmias [1,2]. BrS has an estimated prevalence of one in two thousand, with an increased incidence in the southeast Asian population, and is a leading cause of sudden cardiac death in young, healthy adults [3].

The occurrence of atrial fibrillation (AF) in patients with BrS is higher than in the general population, ranging from 6 to 59% percent [4,5]. AF in the general population increases the risk of stroke, all-cause mortality [6,7], sudden cardiac death (SCD) [8], and other adverse events [9]. Both BrS and AF share mutations in sodium channel function, which may predispose to increased risks of arrhythmias [4]. Knowledge on the impact of AF in the BrS population is still sparse, and a better understanding of it may be of importance for helping the clinicians who manage these patients.

Currently, there is minimal guidance on how to manage patients with concurrent BrS and AF. The gold-standard treatment for patients with BrS is implantable cardioverter defibrillators (ICDs); however, these are only used after assessing a patient’s risk of developing a fatal arrythmia such as VT or VF [10]. Currently, most risk scores do not include concurrent AF as a risk factor [11]. It remains unclear whether concurrent AF increases the risk of fatal arrhythmia in patients with Brugada syndrome.

We aimed to systematically assess the impact of AF in patients with BrS.

## 2. Materials and Methods

We performed a systematic review and meta-analysis of studies enrolling patients with BrS and compared them to patients with BrS and AF in terms of clinical events and genetic background. The review protocol was registered on PROSPERO [12], and the review was conducted in accordance with the Preferred Reporting Items for Systematic Reviews and Meta-Analysis (PRISMA) statement. We did not access or process any patient-identifiable data. Hence, no ethical review or approval was required.

### 2.1. Search Strategy

Searches were run via Ovid MEDLINE, Embase, and Cochrane Cochrane Central Register of Controlled Trials from inception to July 2024, using relevant search terms (“Brugada syndrome”, “atrial fibrillation”, “ventricular fibrillation”, “ventricular tachycardia”, “sudden cardiac death”, “major arrhythmic event”), MeSH terms, and keywords (Annex A in Supplementary Materials). Two reviewers (MAb and MAh) independently screened titles for relevance and then assessed the full texts for their eligibility.

### 2.2. Eligibility Criteria and Population

Studies were considered eligible if they included patients diagnosed with BrS and information on presence/absence of AF. No constraints were set in terms of type of AF. Furthermore, there were no constraints in terms of age, co-morbidities, or any other factors. Studies of patient cohorts implanted with an ICD were excluded.

### 2.3. Outcomes of Interest

The composite primary endpoint of major arrhythmic events (MAEs) was defined as any occurrence of ventricular fibrillation, ventricular tachycardia, sudden cardiac death, and sudden cardiac arrest. Secondary outcomes included syncope, all-cause mortality, and presence of sodium channel mutations.

### 2.4. Data Extraction

The data was extracted onto a pre-made Excel spreadsheet (Office 365, Microsoft Corporation, Redmond, WA, USA) with subheadings for ‘study, number of patients with AF present, number of patients with AF not present, events in patients with AF, events in patients without AF’. The population characteristics were also recorded on an Excel spreadsheet and this included age, percentage of males, BrS type, AF prevalence, follow-up period, country, MAE definition, and type of study (Table 1). Finally, the study type was recorded on the same spreadsheet including the country of the study and if it was single- or multi-centred.

### 2.5. Statistical Analysis

Odds ratios, with 95% confidence intervals, were used as the effect measure. A random-effects model was chosen to account for heterogeneity. The statistical analysis was performed using R (version 4.4.3) (‘meta’ package), with Mantel–Haenszel analysis to create the forest plots [13]. Heterogeneity was assessed using I^2^, and variance (tau^2^) was calculated using the Paule–Mandel model [14].

### 2.6. Sensitivity Analysis

When in the presence of high heterogeneity (I^2^ > 50%), we excluded one study at a time, attempting to detect the potential source of heterogeneity.

### 2.7. Risk of Bias Assessment

The quality of the included papers was assessed using the Newcastle–Ottawa scale (NOS) [15]. This was performed by assessing the selection, comparability, and outcomes of the individual studies, with a maximum score of 4, 2, and 3 in each section, respectively. The scoring was completed by 2 reviewers (MAb and JJHB), and disagreements were resolved by consensus.

**Table 1:** Table of baseline characteristics of the patients and study details.

Study	Takagi 2007 [16]		Kusano 2008 [17]		Cabanelas 2013 [18]		Giustetto 2014 [19]		Calò 2016 [20]		Tokioka 2017 [21]		Asmundis 2017 [22]		Sieira 2017 [23]		Tse 2020 [24]		Honarbakhsh 2021 [25]		Migliore 2022 [26]		Gaita 2023 [27]		Kamakura 2024 [28]	
Characteristic	+AF	−AF	+AF	−AF	+AF	−AF	+AF	−AF	+AF	−AF	+AF	−AF	+AF	−AF	+AF	−AF	+AF	−AF	+AF	−AF	+AF	−AF	+AF	−AF	+AF	−AF
Study design	Prospective cohort		Prospective cohort		Prospective cohort		Prospective cohort		Prospective cohort		Retrospective cohort		Retrospective cohort		Prospective cohort		Retrospective cohort		Retrospective cohort		Retrospective cohort		Prospective cohort		Prospective cohort	
Country	Japan		Japan		N/A		Italy		Italy		Japan		Belgium		Brussels		Hong Kong		International		Italy & Belgium		Italy		Japan	
Single/Multicentre	Multicentre		Single centre		N/A		Multi centre		Multi centre		Single centre		Single centre		Single centre		Multicentre		Multicentre		Multicentre		Multicentre		Multicentre	
No. patients	32	156	10	63	14	264	48	512	18	329	44	202	31	258	31	349	14	261	79	1031	25	80	18	1131	68	345
Age (years)	53		49		43.7		Group 1–47 Group 2–59 Group 3–44		45		47.6		45		41.1		53		51.8		45		45		50.9	
Male (%)	94.7		98.6		N/A		75.7		78.4		95.9		70.2		58.3		90		71.8		75		74		95.6	
Brugada type	Spontaneous or drug-induced type 1		Spontaneous or drug-induced type 1		N/A		Spontaneous or drug-induced type 1		Spontaneous type 1		Spontaneous or drug-induced type 1		Spontaneous or drug-induced type 1		Spontaneous or drug-induced type 1		Spontaneous or fever-induced type 1		Spontaneous or drug-induced type 1		Spontaneous or drug-induced type 1		Spontaneous or drug-induced type 1		Spontaneous or drug-induced type 1	
AF type prevalence (%)	Paroxysmal 17%		Paroxysmal 13.7%		N/A		Persistent & paroxysmal 9%		Persistent and paroxysmal 5.2%		Paroxysmal 17.9%		Paroxysmal 10.7%		N/A		Paroxysmal, persistent & permanent 5%		N/A		Paroxysmal 24%		N/A		N/A	
MAE definition	VF and SCD		VF		VF/polymorphic VT		SCD, VF, and appropriate shock		SCD, aborted SCD, VF, and sVT		SCD, VF, and appropriate shock		SCD and appropriate shock		SCD		VT and VF		SCD, VT, and VF		VT and VF		SCD, VT, and VF		VF	
Follow-up period (months)	Mean 37 ± 16		N/A		Mean 59.3 ± 49.4		Median Group 1–46 Group 2–68 Group 3–41		Mean 48 ± 38.6		Mean 45.1 ± 44.3		Mean 120 ± 55.7		Mean 80.7 ± 57.2		Median 67 (31–113)		Median 63.96 ± 48		Median 155 (128–181)		Median 72 (48–108)		106.8 ± 66.1	

## 3. Results

### 3.1. Characteristics of Included Studies

**Figure 1:**
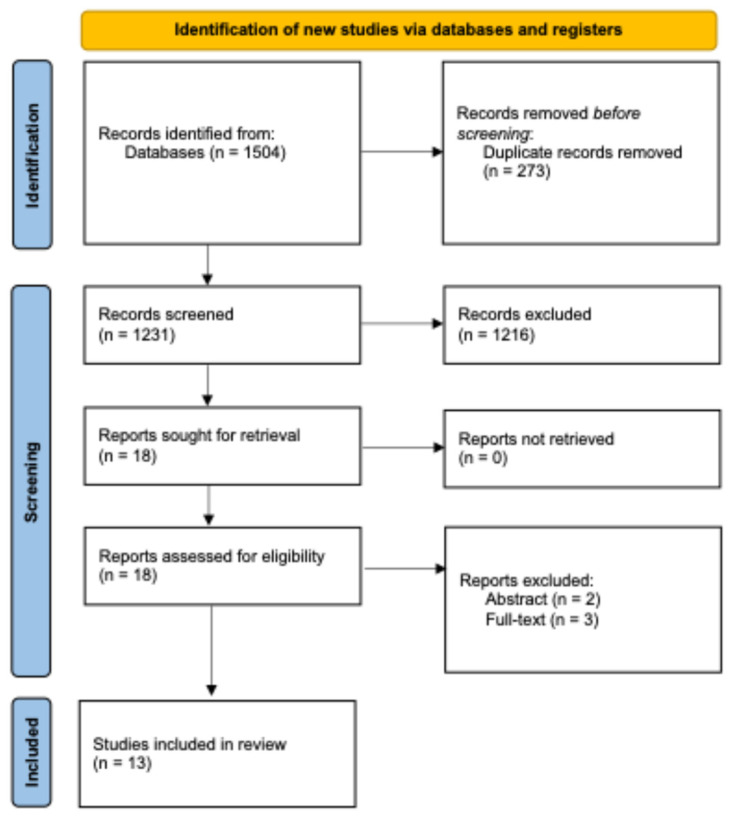
PRISMA flow diagram for reporting the study selection process.

Our searches identified 1504 potentially relevant records. After removing duplicates, 1231 records were screened. After analysing the title and abstracts, the full text was retrieved for 18 records. In the end, 13 studies, including a total of 5413 patients, met the inclusion criteria for this review (Figure 1) [16,17,18,19,20,21,22,23,24,25,26,27,28]. The excluded studies and the reason for exclusion are presented in Table S2 [29,30,31,32,33].

The studies consisted of both retrospective and prospective cohort studies. In total, 432 patients suffered from both BrS and AF and 4981 patients suffered only from BrS.

### 3.2. Study Appraisal

Assessment with the NOS showed that nine studies were considered of a high quality, two were moderate, and two were of a low quality (Table S1; Supplementary Materials).

### 3.3. Meta-Analysis of Extracted Data

**Figure 2:**
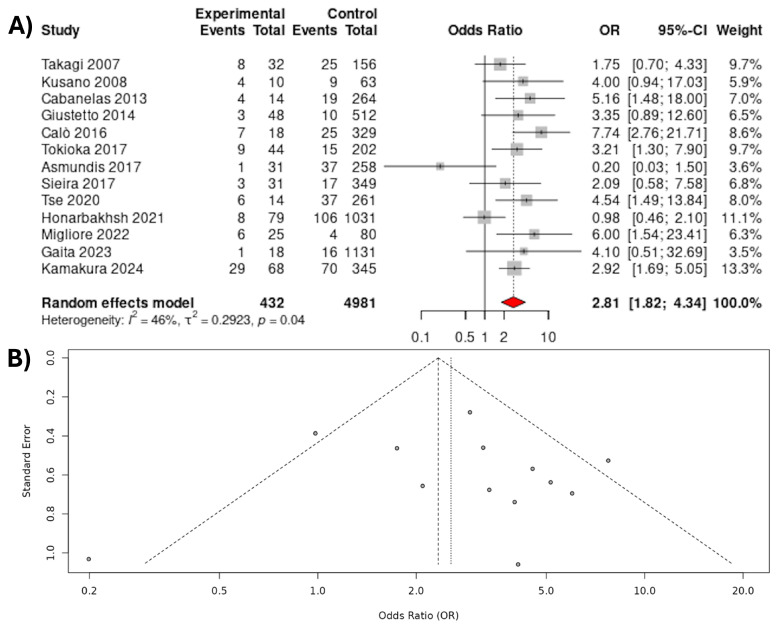
(**A**) Major arrhythmic events in patients with BrS with and without AF—main analysis. (**B**) Funnel plot of major arrhythmic events in patients with BrS with and without AF [16,17,18,19,20,21,22,23,24,25,26,27,28].

An increased rate of MAEs was observed for patients with BrS and AF compared to patients with BrS alone (20.6% vs. 7.8%; OR 2.81, 95% CI 1.82–4.34; *p* < 0.0001; 13 studies). The data had moderate heterogeneity (I^2^ = 46%) (Figure 2). On removal of the Honarbakhsh 2021 study [25] and the de Asmundis 2017 study [22], the heterogeneity dropped to 22.5% and 29.7%, respectively. The results of the leave-one-out analysis can be found in the Supplementary Materials (Table S4).

**Figure 3:**
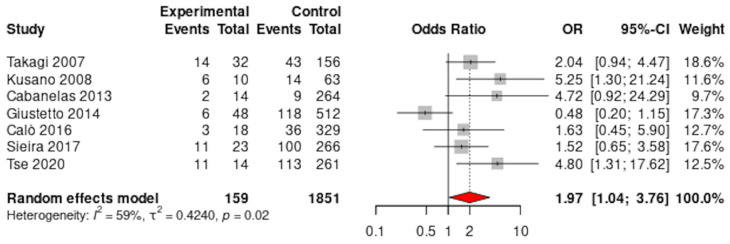
Syncope in patients with BrS with and without AF, [16,17,18,19,20,23,24].

When pooling all of the available data, significant differences in the occurrence of syncope were observed with more events in patients with BrS with AF when compared to BrS without AF (33.3% vs. 23.4%; OR 1.97, 95% CI 1.04–3.76; *p* = 0.04; 7 studies) (Figure 3). There was high heterogeneity for this pooled analysis (I^2^ = 59%), and on removal of the outlier, Giustetto 2014 [19], syncope remained significantly associated with MAEs (OR 2.80, 95% CI 1.66–4.70; *p* < 0.01). Very low heterogeneity was observed (I^2^ = 0%) for this sensitivity analysis (Table S5).

**Figure 4:**
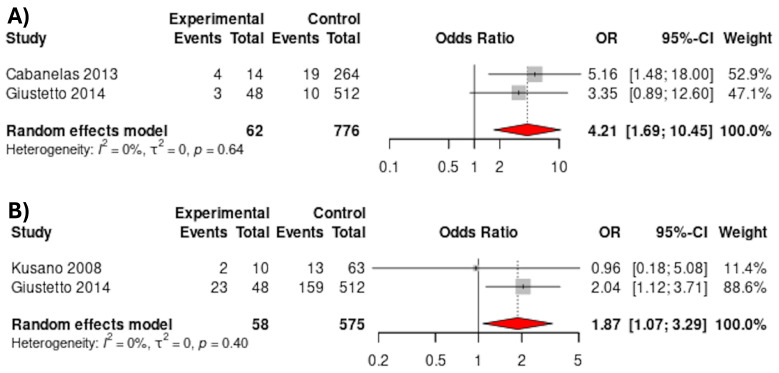
(**A**) Death during follow-up in patients with BrS with and without AF. (**B**) SCN mutations in patients with BrS with and without AF, [17,18,19].

Two studies reported on the association of AF with all-cause mortality [18,19]. Pooling these data suggested that patients with BrS and AF showed higher mortality than patients with BrS alone (11.3% vs. 3.7%; OR 4.21, 95% CI 1.69–10.45; *p* = 0.002) (Figure 4A). The studies showed no heterogeneity (I^2^ = 0%).

Patients with BrS and AF seemed to be more likely to have SCN5a-SCN1b mutations compared to patients with BrS alone (43.1% vs. 29.9%; OR 1.87, 95% CI 1.07–3.29; *p* = 0.028; 2 studies) (Figure 4B). The studies showed no heterogeneity (I^2^ = 0%).

## 4. Discussion

We evaluated the presence of AF as a predictor of clinical events in patients with BrS. The results showed that in patients with BrS, concurrent AF is associated with an increased risk of MAEs. Of the thirteen studies included, Asmundis 2017 [22] and Honarbakhsh 2021 [25] were the only studies that whose effect estimate suggested negative, or lack of, association between patients with BrS and AF and developing MAEs [22]. All of the other studies showed a positive association between AF and MAE [16,17,18,19,20,21,23,24,26,27,28]. These findings aligned with the findings of a previous meta-analysis [34].

When assessing the secondary outcomes, AF was significantly associated with increased rate of syncope and with higher mortality rate during follow-up. These findings suggest that the presence of AF in patients with BrS may potentially be considered a higher risk feature. However, there is uncertainty on whether or not, and in which setting, AF adds incremental risk to other known risk features.

### 4.1. AF Occurrence

Studies have shown broad differences in the reported prevalence of AF in patients with BrS [35,36,37], with most showing a higher prevalence of AF in BrS compared to the general population [38]. Due to the potential prognosis implications of AF in this patient group, it is possible that individuals with BrS may need closer monitoring and screening for AF.

Additionally, the type of AF varied across the studies between paroxysmal, persistent, and unreported. It is possible that the type of AF may result in different rates of arrhythmic events and other secondary outcomes. Research has shown that non-paroxysmal AF is associated with higher risks of death and other significant outcomes [39].

### 4.2. Clinical Implications

After a diagnosis of BrS, the main clinical challenge is to risk stratify and identify patients who are at risk for potential MAE [10]. Currently, patients with BrS who had aborted sudden cardiac death and patients with spontaneous type 1 BrS ECG and cardiac syncope are the ones who are at a higher risk of MAEs [40]. Hence, these two groups patients have an indication for an ICD: class I for the former (secondary prevention) and class IIa for the latter (primary prevention) [41].

The risk of MAEs in patients with spontaneous type 1 Brugada ECG and no symptoms is thought to be 0.5 to 1.0% annually [2,42]. Even though this may seem a low risk at first glance, as most of these patients are young adults, over a lifetime horizon, the true magnitude of risk may be considerable. Hence, more accurate risk stratification is required for identifying those at very low risk, who will have no events, and those with higher chances of MAEs at some point. More risk factors are needed to help clinicians better characterise the risk spectrum. Various risk scores (e.g., BRUGADA-RISK, PAT, Shangai Score System, Sieira et al. [23]), and combinations of multiple risk factors have been proposed for this population [25,43,44,45] (Table S3). Among these, the Shangai Score System includes AF or atrial flutter below the age of 30 as a risk factor [44], and in Sieira et al., only a trend was observed for a potential association between AF and MAE, which resulted in AF not being included in the final model [23]. However, incorporating AF into risk stratification scores must be performed with caution, as the reported prevalence of concurrent AF in patients with BrS ranges from 6 to 59% [4,5]. This means that the addition of AF into risk scores could potentially lead to a large increase in patients deemed eligible for an ICD. This would not only increase healthcare costs but also put more patients at risk of having side effects, such as inappropriate shocks, from a potentially unnecessary ICD. Therefore, future research assessing the incremental role of AF added to traditional risk factors is warranted. On the other hand, the presence of AF also needs to be accounted for when discussing potential disadvantages and complications of ICD therapy with patients, as AF is a common cause of inappropriate ICD shocks, which have an impact on quality of life [43].

Additionally, the management of AF and other supraventricular arrhythmias is complex to manage in patients with BrS. This is due to the contraindications of certain anti-arrhythmic drugs, used to treat AF, in patients with BrS [46]. Furthermore, the presence of AF has been shown to increase the rates of inappropriate shocks from an ICD in patients with BrS [47].

Another option for the treatment of AF in patients with BrS is catheter ablation, particularly pulmonary vein isolation. Studies have shown that ablation in this population has been shown to reduce AF recurrences and decrease the need for inappropriate ICD intervention [48]. Furthermore, it decreases the risk of inappropriate ICD shocks, which can occur when atrial arrhythmias are misinterpreted as ventricular arrhythmias [49]. The most common methods for this are radiofrequency ablation, cryoballon ablation and, more recently, pulsed-field ablation [50]. When comparing the three methods, none has demonstrated overall superiority; however, the use of pulmonary vein isolation has shown to be less successful at preventing recurrences in patients with BrS than those without [47].

### 4.3. Genetics

We observed a significant increase in the rate of SCN5a-SCN1b mutations in patients with both BrS and AF. The SCN5A gene encodes for the pore-forming ion-conducting a-subunit of the sodium channels within the cardiac tissue [51]. The SCN1b gene encodes for the Nav-β1 and -β1B subunits and is linked to atrial and ventricular arrhythmias [52].

The sodium channels are then responsible for the depolarisation of atrial, ventricular, and Purkinje fibres, playing a key role in the initiation and propagation of the cardiac action potential. A loss-of-function mutation in the SCN5A gene results in an imbalance between the inward and outward currents during parts of the cardiac cycle, resulting in an abnormal ECG and arrhythmogenic phenotype, as observed in Brugada syndrome. This has been shown to be the most common genetic association for Brugada syndrome, being observed in 15–30% of BrS occurrences [53]. SCN5A variants have also been associated with AF [54]. Studies have shown that effects from the SCN5A mutation lead to a predisposing environment facilitating AF in patients with BrS [55]. This suggests a shared genetic and molecular basis underling the association between BrS and AF.

### 4.4. Limitations

This systematic review consisted entirely of small observational studies. Heterogeneity was observed for some of the assessed outcomes. We tried to perform sub-analyses and sensitivity analyses to further clarify this. Finally, despite having initially planned to assess the impact of AF in the occurrence of stroke in the BrS population, none of the included studies assessed this endpoint. Due to the young age of patients diagnosed with BrS, reducing the stroke risk via anticoagulation can be clinically challenging due to increased bleeding risk [56]. Therefore, more data is needed to see if traditional risk scores such as CHA2DS2-VASc are appropriate in this patient population.

Additionally, this is a study-level meta-analysis, allowing for no adjustment of covariants for characteristics such as types of AF and type of BrS. Furthermore, the definition of MAEs varies between each study, likely resulting in variation between each patient population. Some studies defined MAEs as VT and VF; some as VT, VF, and SCD; and others as VF alone, while others included inappropriate shocks in their analysis. These variations in the definition of MAEs will likely introduce a level of heterogeneity into the analysis and may affect how the results can be interpreted.

A limitation in our study is the lack of information on strokes as an outcome. Strokes are a major risk in patients with AF and due to the lack of information, we are unable to assess the relationship between the risk of strokes in this patient population, which limits our ability to draw key conclusions on anticoagulation strategies. Furthermore, there was insufficient data to complete a subgroup analysis of the studies.

Some of the studies in this review included retrospective data. This increases the risk of bias such as selection bias and reporting bias. Furthermore, there is variability in the type of Brugada syndrome, including spontaneous and drug-induced, and also variability in the type of AF, including paroxysmal and persistent, which may affect the comparability of the studies.

Finally, it must be noted that AF may not be an independent risk factor. The presence of AF can be associated with other variables, especially genetic factors, making it difficult to draw the conclusion that AF alone is responsible for the observed increased risk.

## 5. Conclusions

Patients with both BrS and AF seem to have a more severe disease phenotype, with a higher rate of MAEs and mortality. More research into the added role of AF in risk stratification of asymptomatic BrS patients is needed, but the prognostic implications of AF may need to be considered when developing future personalised medicine approaches in the BrS population.

## Data Availability

All extracted data from manuscripts are publicly available and have been made available in the tables and plots of the manuscript.

## Supplementary Materials

The following supporting information can be downloaded at https://www.mdpi.com/article/10.3390/jcdd12100391/s1. Table S1: Excluded studies and reasons for exclusion [29,30,31,32,33]; Table S2: Newcastle-Ottawa scale results for included studies [16,17,18,19,20,21,22,23,24,25,26,27,28]; Table S3: Brugada risk stratification schemes; Table S4: Leave-one-out analysis for major arrhythmic events (I^2^); Table S5: Leave-one-out analysis for syncope (I^2^); Annex A: Search Expression.

## Abbreviations

The following abbreviations are used in this manuscript:

AF	Atrial fibrillation
BrS	Brugada syndrome
MAE	Major arrhythmic event
SCD	Sudden cardiac death
