# Supplementary material for: Impact of Atrial Fibrillation on the Outcome of Patients with Brugada Syndrome: A Meta-Analysis
Source: J Cardiovasc Dev Dis. 2025 Oct 3;12(10):391. doi: 10.3390/jcdd12100391 (PMC12564495; doi:10.3390/jcdd12100391)
Supplement: Supplementary file 1 [file jcdd-12-00391-s001.zip › jcdd-3728955-supplementary.pdf]

Supplementary file

Table S1: Excluded studies and reasons for exclusion.

Study id	Reason for exclusion
Gonzalez 2017	Not enough relevant information on patients with concurrent AF
Kawamura 2019	Comparison of procedural outcomes in patients implanted with transvenous and subcutaneous ICDs. Did not assess the outcomes of interest to our study.
Shinohara 2021	Looked into induced VT
Katz 2023	Case report
Bergonti 2024	This paper did not include enough information on patients with AF and their outcomes

References

1. Gonzalez Corcia MC, Sieira J, Sarkozy A, de Asmundis C, Chierchia GB, Hernandez Ojeda J, Pappaert G, Brugada P. Brugada syndrome in the young: an assessment of risk factors predicting future events. Europace. 2017 Nov 1;19(11):1864-1873. doi: 10.1093/europace/euw206. PMID: 27738063.

2. Kawamura I, Nakajima M, Kitamura T, Kaszynski RH, Hojo R, Ohbe H, Sasabuchi Y, Matsui H, Fushimi K, Fukamizu S, et al. Patient characteristics and in-hospital complications of subcutaneous implantable cardioverter-defibrillator for Brugada syndrome in Japan. J Arrhythm. 2019 Sep 16;35(6):842-847. doi: 10.1002/joa3.12234. PMID: 31844476; PMCID: PMC6898525.

3. Shinohara T, Takagi M, Kamakura T, Sekiguchi Y, Yokoyama Y, Aihara N, Hiraoka M, Aonuma K; Japan Idiopathic Ventricular Fibrillation Study (J-IVFS) Investigators. Long-term prognosis in patients with non-type 1 Brugada electrocardiogram: Results from a large Japanese cohort of idiopathic ventricular fibrillation. Ann Noninvasive Electrocardiol. 2021 Jul;26(4):e12831. doi: 10.1111/anec.12831. Epub 2021 Feb 19. PMID: 33608945; PMCID: PMC8293622.

4. Katz A, Balasubramanian S, Freedman Z. Procainamide-Provoked Brugada Pattern in a Patient Presenting with New-Onset Atrial Fibrillation or Flutter: When Does it Matter? J Emerg Med. 2023 Sep;65(3):e229-e233. doi: 10.1016/j.jemermed.2023.04.020. Epub 2023 Apr 26. PMID: 37495422.

5. Bergonti M, Sacher F, Arbelo E, Crotti L, Sabbag A, Casella M, Saenen J, Rossi A, Monaco C, Pannone L, et al. Implantable loop recorders in patients with Brugada syndrome: the BruLoop study. Eur Heart J. 2024 Apr 7;45(14):1255-1265. doi: 10.1093/eurheartj/ehae133. PMID: 38445836; PMCID: PMC10998731.

Table S2: Newcastle–Ottawa scale results for included studies.

Study	Selection	Comparability	Outcomes	Judgement
Takagi 2007	**4**	**1**	**2**	**Moderate**
Kusano 2008	**3**	**1**	**3**	**Moderate**
Cabanelas 2013	**1**	**0**	**1**	**Low**
Giustetto 2014	**3**	**2**	**3**	**High**
Calò 2016	**3**	**2**	**3**	**High**
Tokioka 2017	**3**	**2**	**3**	**High**
Asmundis 2017	**3**	**2**	**3**	**High**
Sieira 2017	**3**	**2**	**3**	**High**
Tse 2020	**2**	**1**	**2**	**Low**
Honarbakhsh 2021	**3**	**2**	**3**	**High**
Migliore 2022	**3**	**2**	**3**	**High**
Gaita 2023	**3**	**2**	**3**	**High**
Kamakura 2024	**3**	**2**	**3**	**High**

Table S3: Brugada risk stratification scores.

BRUGADA RISK

Risk factor	Points
Type 1 BrS ECG pattern in peripheral leads	1
Probable arrhythmia related syncope	1
Early repolarization in peripheral leads	1
Spontaneous type 1 BrS ECG pattern	1

Shanghai Score System

Risk factor	Points
Spontaneous type 1 Brugada ECG pattern at nominal or high leads	3.5
Fever-induced type 1 Brugada ECG pattern at nominal or high leads	3
Type 2 or 3 Brugada ECG pattern that converts with provocative drug challenge	2
Unexplained cardiac arrest or documented VF/polymorphic VT	3
Nocturnal agonal respirations	2
Suspected arrhythmic syncope	2
Syncope of unclear mechanism/unclear etiology	1
Atrial flutter/fibrillation in patients <30 yrs without alternative etiology	0.5
First- or second-degree relative with definite BrS	2
Suspicious SCD (fever, nocturnal, Brugada aggravating drugs) in a first- or second-degree relative	1
Unexplained SCD <45 yrs in first- or second-degree relative with negative autopsy	0.5
Probable pathogenic mutation in BrS susceptibility gene	0.5

PAT

Risk factor	Points
T-peak T-end ≥ 100 msec	5
Arrhythmic or unexplained syncope	5
VT/VF during drug challenge test	4
Prolonged PR ≥ 200 msec	4
Type 1 in peripheral leads	3
aVR sign	3
Fragmented QRS	3
Early repolarization in inferolateral leads	3

Sieira et al

Risk factor	Points
Spontaneous type 1	1
Early familial SCD	1
Inducible EPS	2
Syncope	2
SND	3
SCD	4

Table S4: Leave-one-out analysis.

Leave-one-out analysis for major arrhythmic events (I^2).

Omitting Takagi 2007	48.1%
Omitting Kusano 2008	49.8%
Omitting Cabanelas 2013	47.9%
Omitting Giustetto 2014	50.2%
Omitting Calò 2016	38.5%
Omitting Tokioka 2017	50.1%
Omitting Asmundis 2017	29.7%
Omitting Sieira 2017	50.1%
Omitting Tse 2020	48.4%
Omitting Honarbakhsh 2021	22.5%
Omitting Migliore 2022	47.2%
Omitting Gaita 2023	50.1%
Omitting Kamakura 2024	50.2%
Pooled estimate	45.9%

Table S5: Leave-one-out analysis.

Leave-one-out analysis for syncope (I^2).

Omitting Takagi 2007	65.5%
Omitting Kusano 2008	58.6%
Omitting Cabanelas 2013	62.1%
Omitting Giustetto 2014	0.0%
Omitting Calò 2016	66.1%
Omitting Sieira 2017	65.9%
Omitting Tse 2020	58.6%
Pooled estimate	59.3%

Annex A: Search Expressions.

Search terms:

- "brugada syndrome"[MeSH Terms] OR brugada syndrome[Text Word].

- "atrial fibrillation"[MeSH Terms] OR atrial fibrillation[Text Word].

- "ventricular fibrillation"[MeSH Terms] OR ventricular fibrillation[Text Word].

- "tachycardia, ventricular"[MeSH Terms] OR ventricular tachycardia[Text Word].

- "death, sudden, cardiac"[MeSH Terms] OR sudden cardiac death[Text Word].

- Major arrhythmic event[Text Word].
